# Changes in hemodynamics of pulmonary artery using Flowire in a canine model of acute pulmonary thromboembolism

**DOI:** 10.1002/ame2.70061

**Published:** 2025-07-08

**Authors:** Tomohiko Yoshida, Katsuhiro Matsuura, Akiko Uemura, Ryou Tanaka

**Affiliations:** ^1^ Department of Clinical Veterinary Medicine Obihiro University of Agriculture and Veterinary Medicine Obihiro‐shi Japan; ^2^ Department of Small Animal Clinical Sciences College of Veterinary Medicine University of Florida Gainesville Florida USA; ^3^ Department of Veterinary Medicine Tokyo University of Agriculture and Technology Tokyo Japan

**Keywords:** canine model, pulmonary artery flow peak, pulmonary hypertension

## Abstract

**Background:**

Pulmonary hypertension (PH) is a life‐threatening condition that can be triggered by pulmonary thromboembolism (PTE), which causes abrupt increases in pulmonary artery pressure and resistance. Although Doppler echocardiography is a useful screening tool, its ability to accurately reflect rapid hemodynamic changes during acute PTE remains limited. The Flowire catheter allows for real‐time assessment of intravascular flow and may offer better insight into these changes.

**Aims:**

The aims were to investigate changes in pulmonary artery hemodynamics measured using a Flowire catheter and to validate the accuracy of Doppler echocardiography in assessing these changes in dogs with acute pulmonary thromboembolism (PTE).

**Methods:**

Hemodynamic and echocardiographic data were obtained from 10 anesthetized female beagles using a Flowire catheter and echocardiography at three preload conditions: baseline, bolus loading, and an acute pulmonary hypertension state induced by a 300‐μm dextran microsphere injection.

**Results:**

With increases in pulmonary artery pressure and pulmonary vascular resistance, the proximal and distal pulmonary artery flow peak measured using the Flowire catheter significantly decreased during the acute pulmonary hypertension period. Echocardiography did not accurately capture these hemodynamic changes and tended to overestimate pulmonary artery flow peak in the distal pulmonary artery.

**Conclusion:**

Doppler echocardiography has limitations in accurately reflecting complex hemodynamic changes during acute PTE. In contrast, Flowire catheterization provides additional and precise local hemodynamic information.

## Graphical abstract

This study investigated changes in pulmonary artery hemodynamics using a Flowire catheter and evaluated the accuracy of Doppler echocardiography in dogs with acute pulmonary thromboembolism (PTE). Ten healthy beagles were anesthetized, and acute pulmonary hypertension (PH) was induced by injecting 300‐μm dextran microspheres. Pulmonary artery flow velocities were measured at baseline, after fluid bolus loading, and during acute PTE using both Doppler echocardiography and the Flowire catheter. Flowire measurements revealed significant decreases in both proximal and distal pulmonary artery peak flow velocities after microsphere injection, which corresponded with increases in pulmonary artery pressure and pulmonary vascular resistance. In contrast, Doppler echocardiography failed to capture these hemodynamic changes and tended to overestimate flow in the distal pulmonary artery. Whereas echocardiography and Flowire‐derived flow values showed strong correlation at baseline and during bolus loading, no such correlation was observed during acute PTE. This discrepancy may result from the increased complexity of pulmonary hemodynamics and technical limitations of Doppler imaging under high afterload conditions. Wave reflection from embolic obstruction is believed to contribute to the observed decrease in distal flow velocity. These findings suggest that although Doppler echocardiography is useful for routine evaluation, it has limitations in capturing acute, localized changes in pulmonary artery flow. Flowire catheterization offers a more accurate and detailed assessment of pulmonary hemodynamics in acute PTE models and may be a valuable research tool for evaluating therapeutic interventions or understanding the pathophysiology of PH.

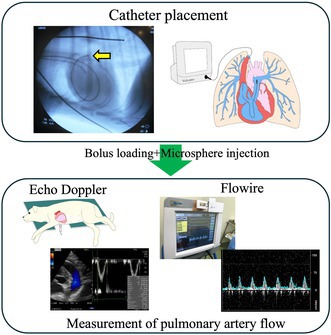

## 1 INTRODUCTION

Pulmonary hypertension (PH) is a pathophysiological condition characterized by thickening and remodeling of the pulmonary arteries due to various causes, leading to increased pulmonary artery pressure (PAP) and pulmonary vascular resistance (PVR).^1^ This condition leads to right‐sided heart failure by increasing afterload on the right ventricle and is associated with a poor prognosis.^2^ Pulmonary hemodynamics can change rapidly in response to pressure overload in pulmonary circulation.^3^ One of the main causes of PH in humans is acute pulmonary thromboembolism (PTE), which often originates from deep vein thrombosis in the lower extremities or pelvis and results in embolic obstruction of the pulmonary arteries.^4^ The development of deep vein thrombosis leads to a sudden increase in PAP, causing significant alterations in pulmonary hemodynamics.^4^ Although two‐dimensional and Doppler echocardiography remain valuable screening tools for the assessment and management of patients with acute PTE,^5^, ^6^ the sensitivity of echocardiography in clinical settings is often limited, reducing its utility as a primary diagnostic method.^7^ Therefore, accurately capturing changes in pulmonary hemodynamics during acute PTE using echocardiography alone remains challenging.^7^ Previous reports have described the use of Flowire to evaluate pulmonary artery hemodynamics in patients with pulmonary embolism.^8^ The Flowire is a catheter‐based guidewire that records intravascular blood flow waveforms in real time and is primarily used to assess coronary and renal blood flow.^9^, ^10^, ^11^ It has also been shown to be useful for evaluating the therapeutic effects of interventions in myocardial and renal infarctions.^12^, ^13^ However, there are a few studies that directly observe and record rapid pulmonary hemodynamic changes in an acute PTE model in dogs using Flowire. Thus, in this study, we investigated the changes in pulmonary artery hemodynamics using Flowire in 10 dogs with acute PTE induced by microsphere injection. We then compared pulmonary artery hemodynamics measured using Flowire with those obtained using Doppler echocardiography.

## 2 MATERIALS AND METHODS

### 2.1 Animal preparation

This study conforms to the ARRIVE guidelines 2.0: updated guidelines for reporting animal research.^14^ All experimental procedures were approved by the Animal Care and Use Committee of Tokyo University of Agriculture and Technology (approval number: R6‐22‐160).

Ten healthy female beagles (Kitayama Labes, Nagano, Japan), aged 12–24 months and weighing 9.1 ± 2.1 kg, were included. Health status was confirmed based on medical history, physical examination, cardiac auscultation, electrocardiography, echocardiography, and standard hematology and serum biochemistry. All clinical and laboratory values were within species‐specific reference ranges prior to experimentation.

### 2.2 Experimental design

**FIGURE 1:**
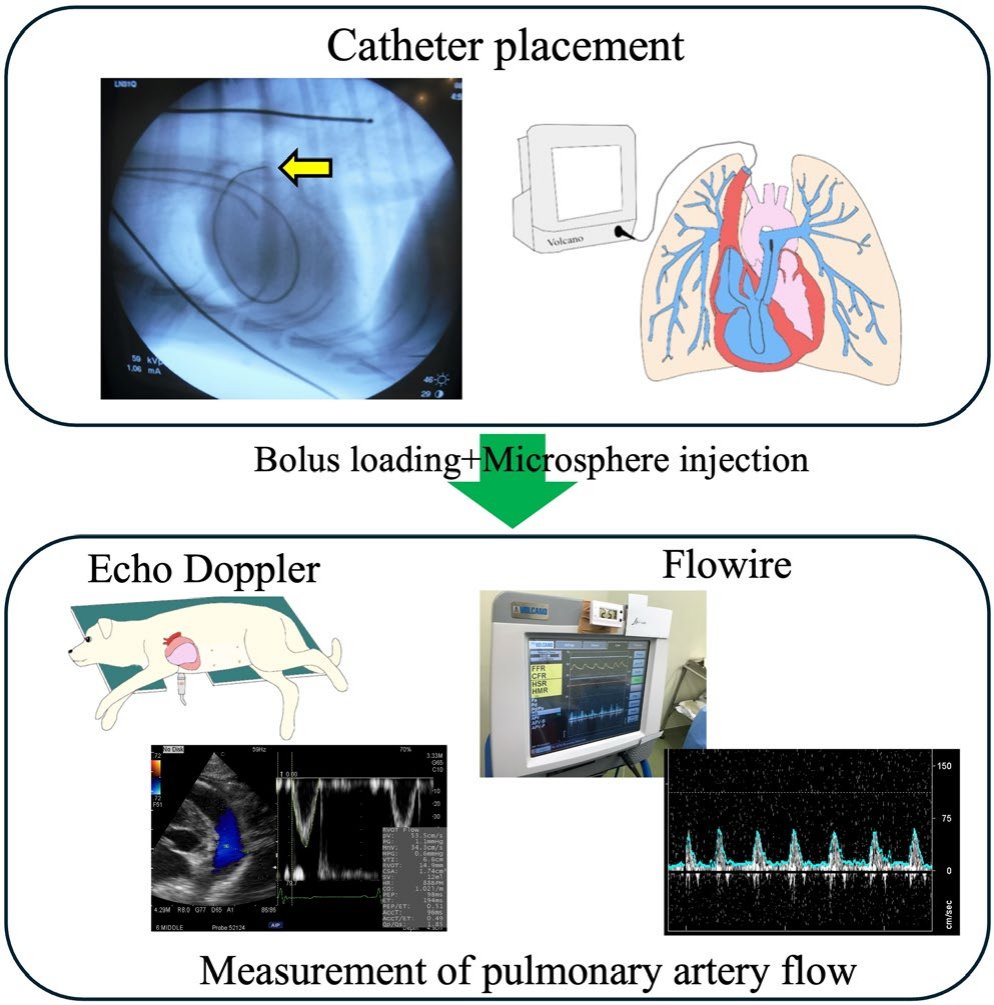
Study design. After catheter placement, echocardiographic and hemodynamic data, including pulmonary artery flow measurements using the Flowire device, were obtained under each loading condition: Baseline, bolus loading, and acute pulmonary hypertension.

All procedures were performed under general anesthesia. This study was designed to record echocardiographic and hemodynamic data, including pulmonary artery flow measurements using a Flowire catheter, in dogs before and after the induction of acute PTE. The method used to induce PTE was based on previously published studies with similar protocols.^14^ To induce PTE and the resulting PH, each dog received intravenous injections of 300‐μm dextran microspheres cross‐linked with epichlorohydrin (Sephadex G‐50, GE Healthcare UK Ltd., England) until mean PAP reached or exceeded 25 mmHg. In addition, to assess the effects of preload augmentation on pulmonary artery flow, each dog underwent bolus fluid loading with lactated Ringer's solution at baseline. The solution was administered intravenously over 5 min at a volume of 100 mL per dog. Hemodynamic and echocardiographic data were recorded under three conditions: (1) baseline, (2) after bolus loading, and (3) during acute PH. Approximately 5 min was allowed for hemodynamic stabilization at each stage. Data were collected in triplicate. An overview of the experimental protocol is shown in Figure 1.

### 2.3 Anesthesia and hemodynamic data acquisition

Each dog was premedicated with buprenorphine (0.02 mg/kg IV, Lepetan, Otsuka Pharmaceutical Co., Ltd., Tokyo, Japan), midazolam hydrochloride (0.2 mg/kg IV, Dormicum, Astellas Pharma Inc., Tokyo, Japan), and atropine sulfate (25 μg/kg IV, Tanabe Seiyaku Co., Ltd., Saitama, Japan). Anesthesia was induced by administering propofol (4 mg/kg IV, Propofol Mylan, Mylan Seiyaku, Tokyo, Japan) and then endotracheal intubation and mechanical ventilation. General anesthesia was maintained with isoflurane (Isoflurane for Animal Use, Intervet, Osaka, Japan) vaporized in oxygen. The end‐tidal isoflurane concentration was maintained at 1.5% ± 0.25%, with peak inspiratory pressures of 10 cm H₂O and a respiratory rate of 8–12 breaths per minute. Heart rate (HR), body temperature, peripheral oxygen saturation (SpO₂), and end‐tidal carbon dioxide (EtCO₂) were recorded every 5 min throughout the procedure.

**FIGURE 2:**
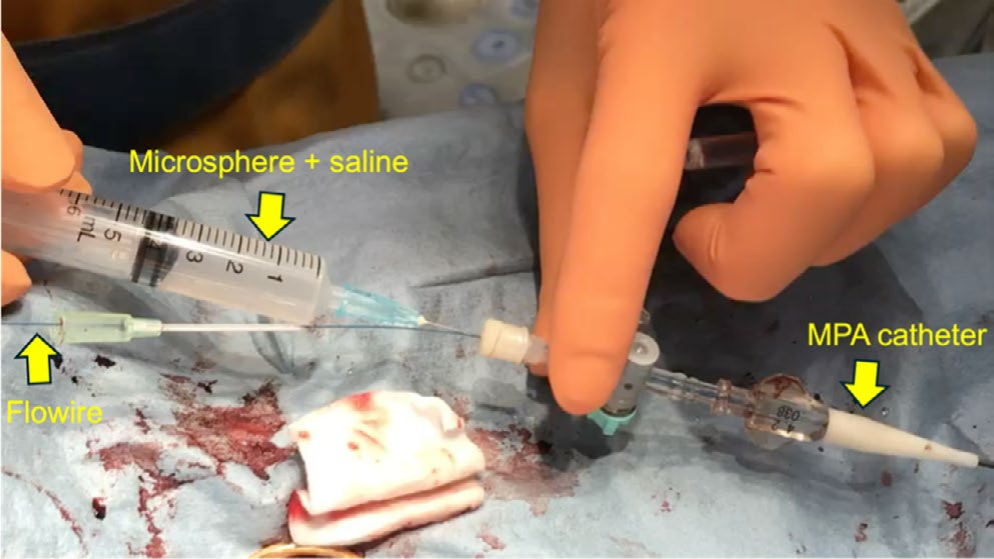
Actual image showing the connection between the multipurpose angiographic (MPA) catheter and the Flowire. The Flowire was inserted into the pulmonary artery via the MPA catheter through the left jugular vein. Microspheres were injected through the MPA catheter.

After the dog was positioned in right lateral recumbency, heparin sodium (100 IU/kg) was administered intravenously to prevent thrombosis. A 4‐Fr catheter (Atom Nutrition Catheter, Atom Medical, Tokyo, Japan) was inserted into the right femoral artery for arterial blood pressure monitoring. A 5‐Fr multipurpose angiographic (MPA) catheter (Goodtec Angiographic Catheter, Goodman, Aichi, Japan) was placed via the left carotid artery and advanced into the left atrium to measure left atrial pressure (LAP). Two additional 5‐Fr MPA catheters were inserted through the left jugular vein: one to monitor right atrial pressure (RAP) and the other to measure PAP, including systolic, mean, and diastolic components. These catheters were connected to pressure transducers (Life Kit DX‐360, Nihon Kohden, Tokyo, Japan), and waveforms were exhibited using a multichannel monitor (Life Scope BSM‐5192, Nihon Kohden). A Flowire (Royal Philips, Amsterdam, the Netherlands) was advanced ~1 cm beyond the pulmonary valve via an MPA catheter inserted through the left jugular vein to record proximal pulmonary artery flow waveforms. After proximal flow was recorded, the Flowire was further advanced 1 cm distally to measure distal pulmonary artery flow waveforms. The catheter–Flowire connection setup is shown in Figure 2. Pulmonary artery flow was recorded for 30–60 s at a sampling rate of 200 Hz (5‐ms temporal resolution). Flow waveforms were smoothed using a Savitzky–Golay filter, and peak flow velocities were averaged over 20 cardiac cycles referenced to the R wave on electrocardiogram (ECG). Cardiac output (CO) was estimated using the Fick method based on oxygen level of blood samples from the left and right atria and measured oxygen consumption.^15^ Stroke volume (SV), PVR, and pulmonary artery compliance (PAC) were calculated as follows:

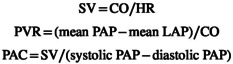




### 2.4 Echocardiographic data

Echocardiography was performed using an Arietta 850 ultrasound system with a 5‐MHz sector probe (Hitachi, Tokyo, Japan). Two‐dimensional, M‐mode, Doppler, and tissue Doppler imaging were conducted according to standard protocols, with simultaneous ECG monitoring. Measurements were obtained from at least five consecutive cardiac cycles during the expiratory phase. Parameters assessed included fractional shortening (FS) and left ventricular internal diameters at diastole and systole, measured from the right parasternal short‐axis view. Right ventricular function was evaluated using tricuspid annular plane systolic excursion (TAPSE) and right ventricular global longitudinal strain (RV GLS) from the apical four‐chamber view. Tricuspid regurgitation (TR) velocity was recorded from multiple angles, with the highest velocity documented. Mitral valve inflow velocities (E and A waves) were measured in the apical four‐chamber view, along with tissue Doppler‐derived early (E′) and late (A′) diastolic velocities at the septal and left ventricular free wall regions. Left ventricular outflow tract velocity was measured using pulsed‐wave Doppler in the apical five‐chamber view. The right ventricular end‐systolic (sRV area) and end‐diastolic areas (dRV area) were measured, and fractional area change (FAC) was calculated as (dRV area − sRV area)/dRV area. Pulmonary artery flow peak velocities were measured from the right parasternal short‐axis view, ~1 cm beyond the pulmonary valve annulus. Acceleration time (AT) was measured from the pulmonary flow profile as the interval from the onset of ejection to peak velocity.

### 2.5 Statistical analysis

All statistical analyses were performed using GraphPad Prism, version 10.0 (GraphPad Software, San Diego, CA, USA). Continuous data are expressed as mean ± standard deviation (SD). Differences among three groups were evaluated using the nonparametric Kruskal–Wallis test and post hoc analysis using Dunn's multiple comparisons test. Differences between two groups were analyzed using Mann–Whitney *U*‐test. Bland–Altman plots were used to assess random and systematic errors between peak pulmonary artery flow velocities obtained using echocardiography and Flowire measurements. Pearson's correlation and linear regression analyses were performed to assess the relationship between echocardiographic and Flowire‐derived pulmonary artery flow velocities. The coefficient of determination (*R* ^2^) was calculated based on the sum of the squared deviations from the regression line. A *p*‐value <0.05 was considered statistically significant.

## 3 RESULTS

### 3.1 Changes in hemodynamic and echocardiographic variables

Changes in hemodynamic variables in the canine model of acute PTE are summarized in Table 1.

**TABLE 1:** Hemodynamic and echocardiographic variables at baseline, during bolus loading, and during acute PH in a canine model of acute pulmonary embolism.

	Baseline	Bolus loading	Acute PH	*p*
Hemodynamic variables				
HR (beats per minute)	126 (86–136)	138 (84–152)	124 (97–138)	0.17
Body temperature (°C)	35.7 (35.1–36.7)	35.65 (35.1–36.8)	35.7 (35.3–37)	0.43
SpO_2_ (%)	99 (96–100)	99 (99–100)	98 (95–100)	0.05
EtCO_2_ (mmHg)	39.1 (28–48)	42 (32–49)	35 (29–42)^†^	0.01^†^
Systolic blood pressure (mmHg)	108 (83–127)	108 (95–143)	96 (72–124)	0.05
Mean blood pressure (mmHg)	87 (60–116)	93 (79–123)	82 (64–118)	0.13
Diastolic blood pressure (mmHg)	87.25 (58–112)	78 (65–132)	71 (58–112)	0.17
Systolic RA pressure (mmHg)	6.6 (3–11)	7 (4–11)	7.8 (4–13)	0.65
Mean RA pressure (mmHg)	4.5 (2–8)	4.5 (2–8)	5 (2–10)	0.94
Diastolic RA pressure (mmHg)	3.3 (0–7)	2.5 (1–7)	4 (1–7)	0.99
Systolic LA pressure (mmHg)	10 (9–17)	11 (10–16)	10.4 (6–14)	0.53
Mean LA pressure (mmHg)	6 (5–12)	7 (6–11)	6 (4–11)	0.43
Diastolic LA pressure (mmHg)	4 (3–9)	4.5 (3–6)	3 (1–10)	0.99
Systolic PA pressure (mmHg)	21 (15–24.3)	22.25 (18–24)	45 (41.6–63)*^†^	<0.01*^†^
Mean PA pressure (mmHg)	14 (10–17)	15 (11–18)	36.5 (28–43)*^†^	<0.01*^†^
Diastolic PA pressure (mmHg)	11.0 (7.44–14)	11.5 (7–15)	32.5 (21.8–38)*^†^	<0.01*^†^
PVR	2.46 (1.67–4.77)	2.25 (1.39–4.12)	18.3 (8.1–26.6)*^†^	<0.01*^†^
Cardio output (L/min)	2.6 (2.2–3.66)	3.205 (2.5–3.66)	1.67 (1.22–2.22)*^†^	<0.01*^†^
Stroke volume (mL)	19 (15–26)	23 (20–29)	13 (2.77–24)*^†^	<0.01^†^
Proximal PA flow peak (Flowire)	84.9 (75.5–127)	93.55 (77–110)	52.5 (44–69.8)*^†^	<0.01*^†^
Distal PA flow peak (Flowire)	80 (70–118)	92 (69–107)	40.5 (34.2–55.1)*^†^	<0.01*^†^
Echocardiographic variables				
FS (%)	33 (24.5–42.6)	42 (32.1–46.7)*	41.6 (34.1–45.2)*	0.01*
LVIDd (mm)	26.99 (21.3–30)	29.5 (24.9–32)	27.87 (23.8–33)	0.28
LVIDs (mm)	18.1 (14.5–20.3)	17.45 (15–19.7)	16.41 (13.9–19.7)	0.28
TAPSE (mm)	11.32 (8.5–13.5)	13.5 (9.2–15.2)	9.1 (7.6–12.5)^†^	<0.01^†^
RV GLS (%)	−17.3 (−30 to 8.9)	−16.2 (−19.3 to 6.3)	−14.54 (−21.5 to 10.1)	0.99
E (cm/s)	46.6 (33–54)	50.75 (28.3–68.1)	39.2 (30.2–56)	0.13
A (cm/s)	44 (18.3–54.6)	49.7 (25.8–66.8)	45.41 (36.7–50.7)	0.13
Sep S′ (cm/s)	6.57 (4.6–9.5)	7.4 (5.9–25.5)	7.1 (4.3–11.5)	0.43
Sep E′ (cm/s)	6.2 (5.1–9)	8.65 (7.7–12.7)*	6.6 (5.2–10.8)*	<0.05*
Sep A′ (cm/s)	5.8 (4–6.7)	8.25 (6–19.1)*	8.12 (4.5–13)	0.04*
Fw S′ (cm/s)	7.22 (5.6–9.5)	9.7 (6.3–12.2)	7.4 (5.6–13.3)	0.35
Fw E′ (cm/s)	7.125 (5–9.8)	8.7 (7.2–13)	7.1 (4.6–12.1)	0.35
Fw A′ (cm/s)	8.1 (6.4–10.5)	9.1 (7.9–17.4)	9.34 (4.5–14)	0.99
LVOT flow (cm/s)	76.05 (37.7–101)	101 (58.4–108.9)	81.1 (64.6–99.1)	0.17
sRV area	1.55 (0.8–2.6)	1.45 (0.52–2.3)	1.56 (1.3–2.5)	0.17
dRV area	2.81 (1.3–5.12)	3.09 (1.03–5.1)	3.694 (2.82–4.4)*	0.02*
FAC (%)	42.2 (19.2–52.8)	51.8 (41.3–60.1)	53.74 (43.18–63.6)*	<0.01*
TR (cm/s)	220 (173.1–247)	247.5 (185–303)	315 (263–433) *^†^	<0.01*^†^
ET (ms)	214 (158–252)	200 (168–267)	197.2 (156–247)	<0.01*^†^
ACT (ms)	96 (56–132)	100 (70–116)	78.2 (56–92)*	0.041*
Proximal PA flow peak (Doppler)	86.6 (71–110)	92.9 (80–105)	81.06 (70.4–91)	0.13
Distal PA flow (Doppler)	83.9 (68–108)	89.5 (77–99)	78.1 (70–88)	0.13

*Note*: Data are expressed as median and interquartile range. Asterisk (*) indicates a significant difference compared to baseline (*p* < 0.05). Dagger (†) indicates a significant difference compared to bolus loading (*p* < 0.05).

Abbreviations: A, late diastolic mitral inflow velocity; A′, late diastolic wave signal as measured using tissue Doppler imaging; ACT, acceleration time; dRV area, right ventricular end‐diastolic area; FAC, fractional area change; E′, early diastolic wave signal as measured using tissue Doppler imaging; E velocity, early diastolic mitral inflow velocity; ET, ejection time; EtCO_2_, end‐tidal CO_2_; FS, fractional shortening; Fw, mitral annulus at the free wall; GLS, global longitudinal strain; HR, heart rate; LA, left atrial; LA, the ratio of the left atrial dimension to the aortic annulus dimension; LVIDd, left ventricular internal dimeter at diastole; LVIDs, left ventricular internal dimeter at systole; LVOT, left ventricular outflow tract; PA, pulmonary artery; PH, pulmonary hypertension; PVR, pulmonary vascular resistance; RA, right atrial; RV, right ventricular; RVEDA index, right ventricular end‐diastolic area index; S′, systolic wave signal as measured using tissue Doppler imaging; sep, mitral annulus at the septal wall; SpO_2_, peripheral oxygen saturation; sRV area, right ventricular end‐systolic area; TAPSE, tricuspid annular plane systolic excursion; TR, tricuspid regurgitation.

Systolic pulmonary artery pressure (sPAP), mean pulmonary artery pressure, diastolic pulmonary artery pressure, and PVR showed no significant changes between baseline and bolus loading periods but were significantly elevated after the injection of dextran microspheres (acute PH). In contrast, CO, SV, EtCO₂, and both proximal and distal pulmonary artery peak flow velocities remained unchanged between baseline and bolus loading but significantly decreased after microsphere injection. FS, septal E′, and septal A′ significantly increased during bolus loading compared to baseline. During the acute PH period, FS, dRV area, and FAC were significantly higher than baseline, whereas TAPSE and pulmonary artery AT were significantly lower. The maximum TR velocity also increased significantly after microsphere injection compared to both baseline and bolus loading.

### 3.2 Effect of acute pulmonary embolism on proximal and distal pulmonary artery flow

**FIGURE 3:**
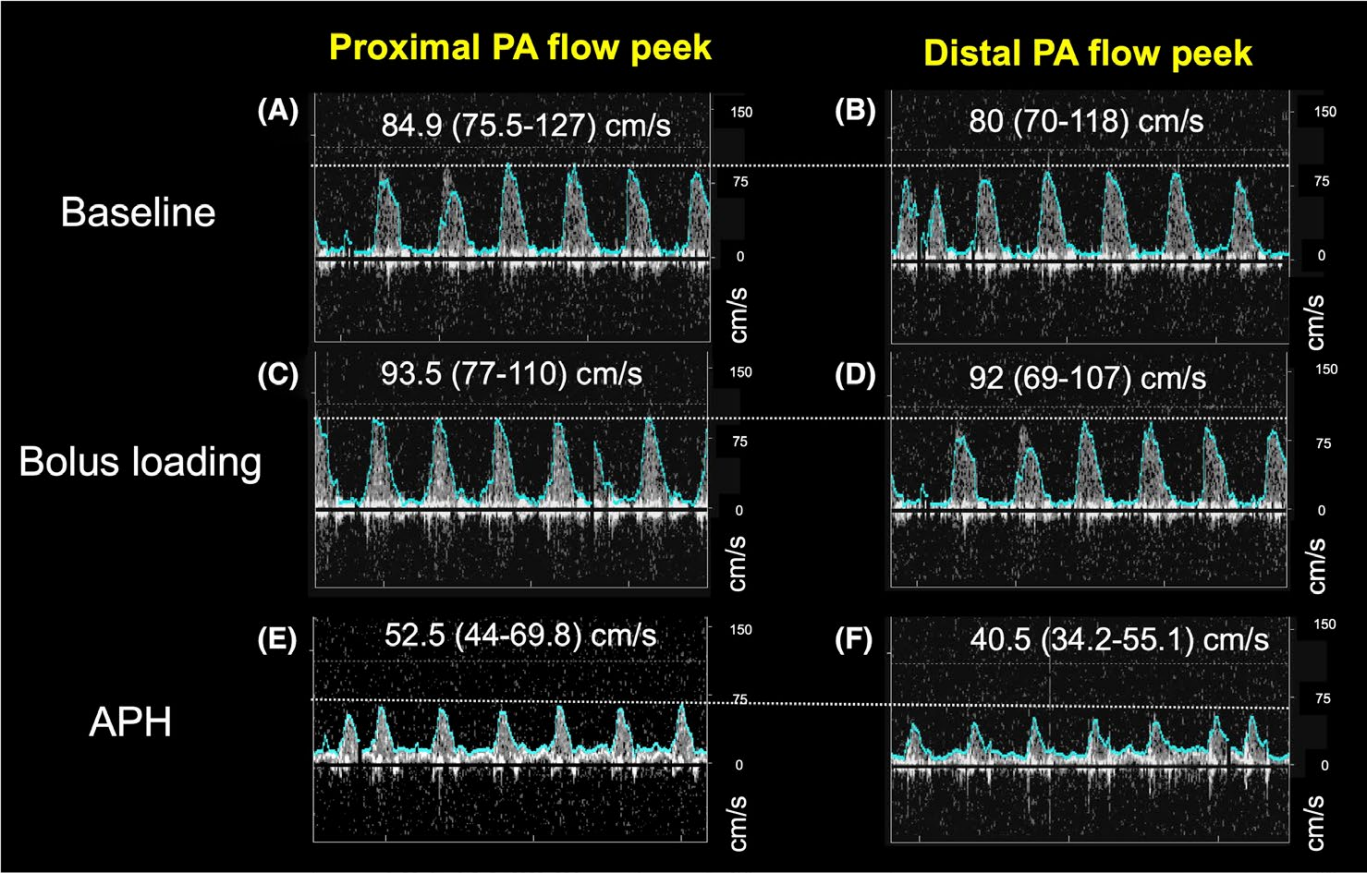
Representative waveforms of pulmonary artery flow obtained using the Flowire device. Waveforms of proximal and distal pulmonary artery flow at (A, B) baseline, (C, D) during bolus loading, and (E, F) during acute pulmonary hypertension. The central values represent pulmonary artery peak flow (median and range, *n* = 10 dogs).

Figure 3 shows representative waveforms of pulmonary artery flow measured using the Flowire in a single dog. During the baseline and bolus loading periods, there were no significant differences between proximal and distal pulmonary artery peak flow velocities (Figure 3A,B: baseline, *p* = 0.25; Figure 3C,D: bolus loading, *p* = 0.48). However, after dextran microsphere injection, distal pulmonary artery peak flow velocity was significantly lower than that of the proximal site (Figure 3E,F: acute pulmonary embolism [APE], *p* < 0.01).

### 3.3 Correlations of distal and proximal pulmonary artery peak flow using echocardiography with that using Flowire

**FIGURE 4:**
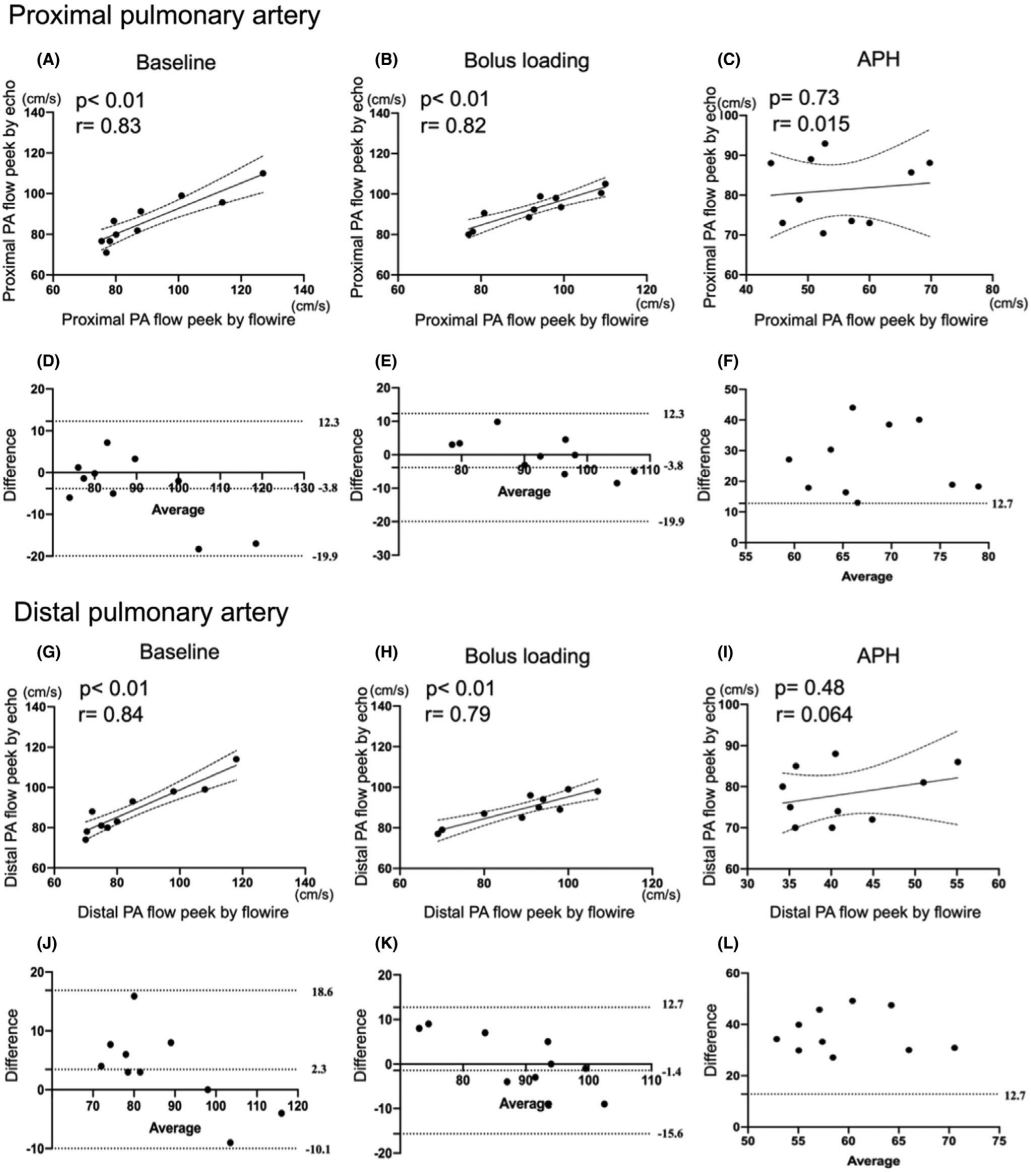
Linear regression and Bland–Altman analyses comparing pulmonary artery peak flow measured using echocardiography and Flowire. The upper figures show the linear regression analyses, and the lower figures show the Bland–Altman analyses comparing pulmonary artery peak flow measured using echocardiography and the Flowire device. (A–F) Data from the proximal pulmonary artery; (G–L) data from the distal pulmonary artery. In artworks A–C and G–I, the solid central line indicates the line of identity, and the dashed lines represent the 95% confidence intervals. In artworks D–F and J–L, the central dashed line indicates the mean difference, and the upper and lower dashed lines indicate the limits of agreement.

Figure 4A–C,G–I show the correlation between distal and proximal pulmonary artery peak flow velocities measured using Flowire and Doppler echocardiography under three conditions: baseline, bolus loading, and acute PH. At baseline and during bolus loading, peak flow velocities measured using echocardiography were linearly correlated with those obtained using Flowire at both proximal and distal sites (Figure 4A,B,G,H). In contrast, during the acute PH period, there was no significant correlation between Flowire‐ and echocardiography‐derived peak flow velocities at either site (Figure 4C,I). Figure 4D–F,J–L present Bland–Altman analyses comparing peak flow velocities obtained using Flowire and echocardiography. During baseline and bolus loading, all data points were within ±2 SDs of the mean difference (Figure 4D,E,J,K). However, during the acute PH period, most points exceeded the limits of agreement (Figure 4F,L). Particularly, Doppler echocardiography tended to overestimate distal pulmonary artery peak flow velocity during this period (Figure 4L).

## 4 DISCUSSION

In this study, we investigated the changes in pulmonary hemodynamics in a canine model of APE using Flowire. The results demonstrated that flow in both the distal and proximal pulmonary arteries decreased during the acute PTE period, which was difficult to detect using echocardiography. However, Flowire effectively captured these changes.

The right heart is known to tolerate volume overload but is significantly more vulnerable to pressure overload. Compared with the left ventricle, the right ventricle demonstrates heightened sensitivity to changes in afterload.^3^ When PH develops, the right ventricle experiences a substantial increase in afterload, which can lead to a significant decrease in CO as well as reductions in pulmonary artery flow velocity.^16^ Our findings are consistent with this, as both PAP and PVR significantly increased after microsphere injection. Although CO and SV initially exhibited compensatory increases, as reflected by contractility indices such as FS and FAC, both values declined after the induction of acute PTE. Previous studies have reported that in patients with chronic PTE, distal pulmonary artery peak flow is significantly lower than proximal peak flow.^8^ This phenomenon is thought to result from wave reflection generated by thrombi, which impedes the forward flow of blood ejected from the heart.^17^, ^18^ In our study, a similar pattern was observed: after microsphere injection, flow velocity in the distal pulmonary artery decreased more significantly than in the proximal artery, likely due to wave reflection caused by the microsphere emboli. Wave reflection occurs not only in relation to PTE but also as a result of pulmonary artery thickening and remodeling, further complicating pulmonary artery hemodynamics.^19^ Castelain et al. have shown that in patients with chronic PTE and primary PH, increased wave reflection in the pulmonary artery significantly affects hemodynamics in both distal and proximal pulmonary vasculatures.^20^ Consistent with these findings, our study demonstrated a significant decrease in distal pulmonary artery flow velocity after microsphere injection, indicating that wave reflection contributes to increased peripheral vascular resistance. This suggests that thrombus‐induced wave reflection plays a key role in altering peripheral vascular resistance.

Another important finding from our study is the discrepancy between the assessment of pulmonary artery peak flow using Flowire and echocardiography after the onset of acute PTE. At baseline and during bolus loading, pulmonary artery peak flow in both the proximal and distal pulmonary arteries measured using Doppler echocardiography significantly correlated with that obtained using the Flowire catheter, with minimal error. However, after the induction of acute PTE, no correlation was observed between pulmonary artery peak flow measured using Doppler echocardiography and that measured directly using the Flowire catheter. One possible explanation for this discrepancy is that pulmonary hemodynamics becomes so complex during acute PTE that accurate assessment using echocardiography becomes difficult. In addition, there are technical limitations to the noninvasive assessment of pulmonary artery peak flow using Doppler echocardiography. Factors such as measurement angle, probe distance, respiratory variability, and operator‐related error can all contribute to inaccuracies in Doppler‐based evaluations.^21^, ^22^ Previous studies have reported that although the waveform of pulmonary artery flow measured using Doppler echocardiography changed in patients with PH, its peak velocity did not change.^23^ This finding is consistent with our results, in which Doppler‐based assessments failed to capture the decrease in pulmonary artery peak flow during the acute PTE period. In contrast, catheter‐based measurements, such as those obtained using Flowire, have been shown to more accurately reflect changes in pulmonary artery peak flow under conditions of increased afterload.^8^ Given the inherent limitations of Doppler echocardiography in accurately capturing complex changes in blood flow, especially during acute PTE, catheter‐based measurements provide a more precise method for assessing pulmonary artery hemodynamics. This is particularly important because pulmonary artery peak flow varies significantly between measurement sites due to the influence of wave reflection. Whereas Doppler‐based assessment of pulmonary artery flow velocity has certain limitations, our study demonstrated that the estimated SPAP, calculated using the formula 4 × (TR velocity max)^2^ + RAP, closely matched the catheter‐measured SPAP, with both averaging ~45 mmHg. This agreement may enhance the reliability of echocardiography‐derived estimates of PAP, particularly in clinical settings where invasive measurement is not feasible. Although parameters such as PAP and PVR estimated using echocardiography are widely used to evaluate the severity of PH,^24^ incorporating detailed assessments of pulmonary artery flow using Flowire may offer new insights into pulmonary hemodynamics and improve the accuracy of clinical evaluations.

## 5 STUDY LIMITATIONS

This study established an animal model of PH that mimicked acute thromboembolic PH by embolizing the peripheral pulmonary arteries with microspheres. However, the hemodynamic characteristics of this model may differ substantially from those observed in patients with pulmonary arterial hypertension or chronic thromboembolic disease typically encountered in clinical settings. In addition, the sample size in this study was small, and the results may have varied depending on the number of dogs included. Although the placement of the Flowire catheter was standardized across all cases using fluoroscopic guidance, there remained the possibility of displacement due to respiratory movement or technical errors. Furthermore, this study utilized young dogs only, without accounting for age‐related changes in pulmonary hemodynamics, which may limit the generalizability of the findings.

## 6 CONCLUSION

In a canine model of acute PTE, substantial alterations in pulmonary hemodynamics were observed, specifically a decrease in pulmonary artery peak flow in both the proximal and distal pulmonary arteries. Whereas these acute changes in pulmonary artery flow were detectable using the Flowire catheter, they were not captured using Doppler echocardiography. Due to the complex nature of pulmonary circulatory changes after the onset of PH, it is essential to evaluate the condition comprehensively using multiple parameters, including PAP, PVR, and changes in pulmonary artery peak flow.

## AUTHOR CONTRIBUTIONS

**Tomohiko Yoshida:** Conceptualization; formal analysis; writing – original draft; writing – review and editing. **Katsuhiro Matsuura:** Formal analysis; investigation; validation. **Akiko Uemura:** Conceptualization; writing – original draft. **Ryou Tanaka:** Conceptualization; supervision; writing – review and editing.

## FUNDING INFORMATION

This study was partly supported by a Grant‐in‐Aid for Scientific Research (JSPS KAKENHI 24K18010). The authors confirm that the funding agency had no role in the study design, data collection and analysis, manuscript content, or the decision to submit the article for publication.

## CONFLICT OF INTEREST STATEMENT

The authors declare no conflict of interest related to this study.

## ETHICS STATEMENT

All procedures involving animals were conducted in accordance with the guidelines of the the Animal Experimental Committee of Tokyo University of Agriculture and Technology and were approved by the Animal Ethics Committee of the university (Approval No. 30‐146).

## Data Availability

The datasets generated and/or analyzed during the current study are available from the corresponding author on reasonable request.
